# Managing Massive Splenic Hematoma: Exploratory Laparotomy or Active Surveillance?

**DOI:** 10.7759/cureus.14967

**Published:** 2021-05-11

**Authors:** Steve B Otieno, Saradasri K Wellikoff, Alva Weir, Osarenren Ogbeide

**Affiliations:** 1 Internal Medicine; Hematology/Oncology, The University of Tennessee Health Science Center, Memphis, USA; 2 Internal Medicine; Hematology/Oncology, Veterans Affairs Hospital, Memphis, USA

**Keywords:** splenic hematoma, abdominal trauma, non-operative management

## Abstract

Splenic hematoma is a known complication of blunt force abdominal trauma. Traditional management of splenic hematomas has been primarily surgical. However, more recently, spleen-sparing management has been favored over surgical management for cases that meet certain criteria, with surgery now reserved for patients with complications. In this report, we present a case of a massive splenic hematoma that was managed conservatively and analyze the challenges faced in clinical decision making.

## Introduction

The spleen and liver are commonly injured in cases of blunt force abdominal trauma such as motor vehicle accidents [1-2]. Splenic injuries can be fatal, not only due to hemorrhage from the trauma but also from the delayed rupture of sub-scapular hematoma or pseudoaneurysms [3]. In recent decades, nonoperative management has been favored in treating splenic injuries over surgical management with the objective of preserving the organ and its functions. Apart from the attendant problems related to any laparotomy procedure, splenectomy is additionally associated with the significant morbidity of lifetime risk of overwhelming sepsis, particularly from encapsulated organisms. Nonoperative management of splenic trauma was first proposed by Upadhyaya and Simpson in 1968 [4]. Gradually, this approach has gained widespread acceptance, initially in pediatric surgery, and more recently in adult trauma care. The decision to choose either nonoperative or surgical management is usually driven by several criteria including but not limited to the degree of anatomic injury to the spleen and other associated structures as well as the patient’s hemodynamic stability [3].

In this report, we discuss a case of a 66-year-old patient who initially presented with abdominal pain, weakness, and anemia. This case presented a significant dilemma to us due to inaccurate history and poor recollection of events on the part of the patient, ultimately leading to his massive splenomegaly.

## Case presentation

A 66-year-old male with a past medical history significant for hypertension, diabetes, coronary artery disease, hepatitis C, benign prostatic hypertrophy, polysubstance abuse, and post-traumatic stress disorder with suicidal ideation presented to the Veterans Affairs Hospital's Emergency Department with abdominal pain, weakness, and anemia. He denied any history of falls or abdominal trauma.

On initial evaluation, his vital signs were as follows: a blood pressure (BP) of 148/78 mmHg, heart rate (HR) of 122 beats per minute, respiratory rate (RR) of 16 breaths per minute, and temperature (T) of 97 ^o^F. Physical examination revealed a full, soft abdomen with no masses and normal bowel sounds. Laboratory studies showed mild leukocytosis, microcytic anemia with a hemoglobin of 6.7 g/dL, hematocrit levels of 25.1%, and thrombocytosis with a platelet count of 860 x 10^9^/L. The chemistry panel was normal. Prothrombin time of 16.8 and activated partial thromboplastin time of 35.1 were noted. Chest X-ray showed a left lower lobe opacity suggestive of consolidation or pleural effusion. He was admitted for pneumonia and anemia.

A subsequent CT scan of the abdomen showed a very large (16 x 14 x 13 cm), complex cystic mass in the left upper quadrant (LUQ), epicentered in the region of the spleen, with peripheral enhancing nodularity. This was suggestive of a large necrotic mass, presumably within the spleen. No normal splenic tissue was seen. There was also a moderate-sized left pleural effusion with left lower lobe compressive atelectasis. The patient received intravenous fluids and blood transfusion and was started on a pain-management regimen. An abdominal ultrasound revealed a large (16 x 12 x 15 cm) solid mass in the LUQ.

Surgery, Gastroenterology, and Hematology/Oncology services were consulted. The patient again denied any history of falls, blunt force trauma, or abdominal injury. He did report a poor appetite and weight loss. He also informed that he had prior hospitalizations in other area hospitals for similar complaints and had undergone a colonoscopy at one hospital, which had revealed internal hemorrhoids but no bleeding. CT angiography at the previous hospital had revealed no bleeding but had shown splenic, hepatic, and renal cysts. The case was discussed in the tumor board, where it was determined that the mass was not consistent with a hemorrhage. Working diagnoses included a pseudocyst, a complex splenic cystic, a hemorrhagic splenic cyst, a cystic splenic neoplasm, or a parasitic infection. The plan was to perform thoracentesis and send the pleural fluid for cytology and analysis for infection. The surgical recommendation was that he would likely need a splenectomy, and immunization was recommended.

Thoracentesis was performed with the analysis of the pleural fluid revealing an exudative effusion with 2+ polymorphonuclear neutrophils with no organisms seen. Cytology was negative for malignancy. An esophagogastroduodenoscopy with endoscopic ultrasound (EUS) showed a gastric polyp, which was biopsied with pathology and revealed minimally inflamed mucosa; there was no dysplasia or malignancy and it was negative for *Helicobacter pylori*. The EUS showed a large, 14-cm LUQ mass, with some hyper-echoic areas within the mass, which raised concerns about intra-tumoral bleeding or necrosis.

In light of the above-mentioned findings, a continuation of the conservative approach was pursued, and the patient's condition gradually began to improve. He was able to tolerate a diet; the pain became less severe and his counts were stable.

We reviewed all of the patient’s imaging radiology, and on a closer look at the scans, the initially reported findings of “splenic cysts” were determined to be more suggestive of a contained splenic bleed. On further direct and focused questioning of the patient, it was revealed that he had indeed sustained a fall on his left side a few weeks prior to the development of his symptoms.

**Figure 1:**
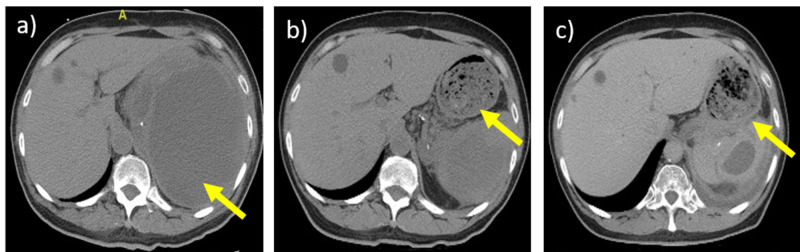
Evolution of the splenic hematoma The yellow arrows show the spleen and changes in the hematoma at a) day 1, b) day 32, and c) day 67

A repeat CT scan showed a decrease in the size of the cystic lesion from 14 x 13 x 16 cm to approximately 8.6 x 8.1 x 10.5 cm. A follow-up CT scan three weeks later showed that the mass had now decreased to a size of 4.8 x 3.0 x 4.8 cm. The report on this last scan also noted that “the spleen overall is somewhat irregular in shape and in density suggesting this may represent a resolving traumatic injury to the spleen.” Figure 1 shows the evolution of the splenic hematoma. The patient did not undergo a celiotomy and has continued to show improvement; he is currently being followed up in the clinic for delayed complications of the nonoperative management of his blunt force splenic injury.

## Discussion

As previously noted, there has been a shift in the approach to the management of splenic injury following abdominal blunt force trauma in recent years [5,6]. With the improvement in imaging techniques and the ability to carefully monitor patients, there has been a decline in surgical management. Several criteria have been proposed by various groups to assist with triaging patients in the immediate post-injury period and during ongoing active surveillance. Hemodynamic stability with minimal resuscitation, younger age, no alteration of mental status, documentation of splenic injury by imaging, and absence of serious associated intra-abdominal injury are some of the traditional assessment criteria. The issue of the patient's age has been explored, and some have advocated the avoidance of a nonoperative approach in those over the age of 55. In addition, the limitations of CT and other imaging modalities in assessing splenic, hepatic, and associated hollow viscus injury have been raised [7]. The Buntain classification of splenic trauma, which grades it on a scale of 1 to 6, has been proposed as a viable way to differentiate those who should be approached surgically versus conservatively [8]. Our patient was somewhat atypical in the sense that he presented with a large spleen and anemia and raised high suspicion for recent splenic trauma and bleed, likely a few weeks prior to his arrival at our facility. Our patient’s management of blunt trauma to the spleen was not by design but more circumstantial in nature.

A retrospective study conducted at the University of Texas at Houston, a level 1 trauma center, has provided some insight into the delayed complications of nonoperative management of blunt adult splenic trauma [5]. The study involved 280 patients who were admitted with blunt force splenic injury; 66% were men, and the mean age was 32.2 years. Of note, 21% died of multiple injuries within 48 hours and 134 (48%) were treated operatively within the first 48 hours, and 87 patients (31%) were managed nonoperatively. Patients who were managed nonoperatively had significantly lower injury severity scores compared to those managed operatively. The injury severity score is an established tool for assessing the severity of trauma. Length of stay was lower in the operative group while the use of blood products was significantly higher. Seven patients (8%) who were managed nonoperatively developed delayed complications requiring intervention. Five had overt bleeding, which occurred at four days for three patients, at six days for one patient, and at eight days for one patient. This is in line with earlier reports where bleeding was reported early in the period of observation. Three patients underwent splenectomy, one had a splenic artery pseudoaneurysm embolization, and one patient had embolization to two areas of bleeding. Two had delayed splenic abscesses at approximately one month and were both treated by splenectomy.

## Conclusions

This case highlights the aspects of conservative management of late complications of splenic injury from blunt force abdominal trauma. As noted above, patients who have severe injuries with either overt or occult bleed can become symptomatic shortly after the injury or within the first week to 10 days. Subsequently, many patients do reasonably well with serial observation, monitoring for late complications, and nonsurgical management as described in this case report.
